# Unraveling the Role of Empathy and Critical Life Events as Triggers for Social Entrepreneurship

**DOI:** 10.3389/fpsyg.2020.579500

**Published:** 2020-11-06

**Authors:** Wim Lambrechts, Marjolein C. J. Caniëls, Ingrid Molderez, Ronald Venn, Reinke Oorbeek

**Affiliations:** ^1^Department of Marketing and Supply Chain Management, Faculty of Management, Open Universiteit, Heerlen, Netherlands; ^2^SEIN—Identity, Diversity & Inequality Research, Faculty of Business Economics, Hasselt University, Hasselt, Belgium; ^3^Department of Organization, Faculty of Management, Open Universiteit, Heerlen, Netherlands; ^4^Centre for Economics & Corporate Sustainability (CEDON), KU Leuven - Leuven University, Campus Brussels, Brussels, Belgium

**Keywords:** empathy, social entrepreneurship, life event, social impact, hybrid approaches

## Abstract

Social entrepreneurs are generally believed to have started their venture to improve societal needs and create social value. Yet, in order to achieve continuity of their organization, they need to generate economic value as well. These seemingly opposite objectives of social and economic value creation can cause tensions in social enterprises. This study aims to derive in-depth insights into personal dispositions and motivations of social entrepreneurs, with a specific focus on empathy. The study assesses differences in motivations of social entrepreneurs and how moral empathy helps them to cope with tensions that arise from trying to achieve both commercial and social goals. Analysis of semi-structured interviews with 33 social entrepreneurs in Belgium explores the tension between social and economic objectives as a paradox social entrepreneurs have to deal with. First, empathy is an important motivator, but not for all entrepreneurs. Social entrepreneurs who are driven by empathy often indicate that experiencing a critical life event has led to certain business choices. The life event does not always directly lead to increased empathy but often changes an entrepreneur’s career or life path. Second, while social entrepreneurs are supposed to stress social impact, some respondents firmly state that financial impact is more important to their organization. The results show that social entrepreneurs display other motivations that are typical for “traditional” (commercial) entrepreneurs as well, such as self-realization and the sense of doing meaningful work. Empathy seems to play an important role in successfully dealing with the paradox and tensions between social and economic value creation, and more specifically to prevent mission drift.

## Introduction

In recent decades, social entrepreneurship has been developing as a research field focusing on the role and interactions between government, non-governmental organizations (NGOs) and profit-focused businesses (Bacq and Janssen, 2011; Guo et al., 2020). Diminishing government support encourages entrepreneurs to tackle emerging societal problems in an innovative and sustainable manner (Dwivedi and Weerawardena, 2018; Lubberink, 2020). Contemporary examples in the literature date back to the late 1960s and include the Ashoka Foundation, which was started in India, providing seed capital to entrepreneurs with a social vision; and the Grameen Bank, which was established to fight poverty in Bangladesh (Mair and Martí, 2006). However, social entrepreneurship predates to the 20th century (Molderez, 2020). New Lanark Mills is often seen as the first example of social entrepreneurship (Shaw and Carter, 2007). It was founded by David Dale in 1786 and afterward successfully managed by Robert Owen in the early 1800s. Rabello et al. (2018) even date social entrepreneurship to the 17th century, when housing programs for workers were set-up.

Over the past decades, social entrepreneurship has attracted increasing interest from scientists, as evidenced by the growing number of publications about social entrepreneurship (Roper and Cheney, 2005), especially after 2010 (Dacin et al., 2011; Smith et al., 2013; Stephan and Drencheva, 2017). Being a nascent field, research about social entrepreneurship is not without challenges. Dacin et al. (2011) refer to developing new insights into existing theories. In their entrepreneurship research, Schumpeter (1934) and McClelland (1961) adopted a psychological perspective. They focused on entrepreneurs as individuals with personal motivations (Frese and Gielnik, 2014). Recent studies confirm that entrepreneurship and entrepreneurial success are driven by personal motivations (Baum et al., 2014; Frese and Gielnik, 2014). Consequently, the question arises about which personal motivations drive social entrepreneurship. This question has been addressed in several studies which portray mixed outcomes (Bargsted et al., 2013; Smith et al., 2014; Bacq et al., 2016; Yitshaki and Kropp, 2016; Stephan and Drencheva, 2017).

In an overview study, Stephan and Drencheva (2017) show that social entrepreneurs are similar to “traditional” entrepreneurs in terms of their key characteristics. However, social entrepreneurs differ from traditional entrepreneurs by having more “social concerns,” a greater sense of moral obligation and a higher degree of empathy (Miller et al., 2012; Bargsted et al., 2013; Ruskin et al., 2016; Lubberink, 2020). Most of the studies report on (nascent) entrepreneurs who have the intention to engage in social (e.g., Saebi et al., 2018; Wang et al., 2019; Wu et al., 2020) or sustainable (e.g., Ploum et al., 2019) entrepreneurial activities. More empirical research is needed that concentrates on entrepreneurs who already have started their venture and actually create social value in combination with an economic activity. In-depth analysis is required to uncover all motives upon which entrepreneurial activities are built (Zahra et al., 2014). Stephan and Drencheva (2017) emphasize the insufficiency of research with respect to the role of contextual factors in the personality and motivation of social entrepreneurs. Despite the different interpretations of contextualization (Zahra et al., 2014), this article uses a very specific meaning of the term, i.e., the personal context of the social entrepreneur, which is an unexplored dimension of Zahra et al. (2014). This focus helps to understand the start, practices, and management of social entrepreneurs and pays attention to even the smallest motives for entrepreneurial activity.

Personal motivations of social entrepreneurs are essential to the development of their activities and focus on social impact. It is suggested that social entrepreneurs are characterized by a relatively high degree of moral empathy, but evidence is limited and sometimes contradictory (Stephan and Drencheva, 2017). Chandra and Shang (2017) found that motivations can be traced back to a social entrepreneur’s past. An important “life event” can trigger a traditional entrepreneur to focus on a social cause (Shumate et al., 2014). Furthermore, unique personal circumstances are suggested as a reason to start a social enterprise (Saebi et al., 2018). Stephan and Drencheva (2017, p. 215) indicate the need for further research into situational triggers that prompt individuals “to act on longstanding motivations and values.” Personal values play an important role in the creation of an enterprise. Depending on the priority that is given to specific values, the type of enterprise will be different (Conger, 2012). Since values are shaped by “individual heritage and experience” (Conger, 2012, p. 92), it is relevant to look behind elements that shape experience, such as personal events.

This study fills these gaps in current understanding by focusing on typical motivations of social entrepreneurs, the role of moral empathy in motivations of social entrepreneurs, and how critical life events play a role in developing empathy. Data is gathered *via* semi-structured interviews from 33 Belgian social entrepreneurs. This study makes two major contributions to the existing literature about social entrepreneurship. First, the research design addresses the shortcomings of prior studies, which predominantly are conceptual in nature (e.g., Miller et al., 2012; Arend, 2013; Grimes et al., 2013), target social entrepreneurship intentions instead of actual ventures (e.g., Hockerts, 2017; Bacq and Alt, 2018), or focus mainly on heroic portraits of social entrepreneurs (Stephan and Drencheva, 2017). Semi-structured interviews with social entrepreneurs allow to gather in-depth information about contextual factors and motivations that have induced the social entrepreneurial initiative. Second, this study extends current knowledge about situational factors, such as life events, that trigger individuals to act on deep-rooted motivations and personal values. Whereas only a few prior studies on specific entrepreneurial motives have pointed toward important drivers in the personal environment of a social entrepreneur, this study will explicitly address these contextual factors. Without a good understanding of contextual factors that influence an entrepreneur’s choices, it is difficult to shed light on the true motivations of social entrepreneurs.

## Literature Review

### Social Entrepreneurship

The academic literature focusses on how social entrepreneurship differs from “traditional” (commercial) entrepreneurship. Different definitions of social entrepreneurship can be found. The diversity and heterogeneity of social enterprises explain the absence of a universally accepted definition. The co-existence between the public sector and the traditional private sector has led to naming them the third sector (Shaw and Carter, 2007). According to Alter (2003), they are part of the hybrid sector, combining social, and business interests. Benefit Corporation (B-Corp) entrepreneurs are a recent example of commercial enterprises that incorporate social value creation (Roth and Winkler, 2018). Peredo and McLean (2006, p. 64) define social entrepreneurship as follows: “Social entrepreneurship is exercised where some person or group: (1) aim(s) at creating social value, either exclusively or at least in some prominent way; (2) show(s) a capacity to recognize and take advantage of opportunities to create that value (“envision”); (3) employ(s) innovation, ranging from outright invention to adapting someone else’s novelty, in creating and/or distributing social value; (4) is/are willing to accept an above-average degree of risk in creating and disseminating social value; and (5) is/are unusually resourceful in being relatively undaunted by scarce assets in pursuing their social venture.” This definition highlights entrepreneurship in the sense of value creation, as well as the social aspect of this value creation. Responding to the need of operationalizing social entrepreneurship, Dwivedi and Weerawardena (2018) concluded on the basis of a large scale survey that five dimensions are important. According to their research, effectual orientation is the strongest indicator, followed by social mission orientation, innovativeness, risk management, and proactiveness.

Mair and Martí (2006, p. 36) emphasize the difference between a “traditional” entrepreneur and a social entrepreneur: “Social entrepreneurship is seen as differing from other forms of entrepreneurship in the relatively higher priority given to promoting social value and development versus capturing economic value. (…) Social entrepreneurs are individuals who start, lead, and manage organizations that seek to create social value by addressing societal challenges such as environmental degradation, ill health or social exclusion.” In general, definitions of social entrepreneurship stress that the distinguishing feature of a social enterprise is the dual mission of creating social value on top of economic value (Saebi et al., 2018). Social enterprises are characterized by creating social value, but that does not imply that all social value creating organizations are social enterprises (Dwivedi and Weerawardena, 2018). More and more commercial organizations create social value, but their motive remains profit-oriented. The distinctive feature for social organizations, however, remains their social mission motive (Alter, 2003; Dacin et al., 2011; Stephan and Drenchava, 2017; Dwivedi and Weerawardena, 2018). Having a mission of creating social value does not contradict a business interest. According to Dacin et al. (2011), creating an economic value is crucial for the viability of the organization. The income they receive is reinvested in the organization.

Given the duality in their mission, social entrepreneurs may be at risk of losing sight of their social mission when pursuing revenue (Ebrahim et al., 2014; Maier et al., 2016). There are many examples of organizations that were founded on empathic and people-oriented motivations. For various reasons, they ended up being carried along a different path, i.e., prioritizing economic activities over social objectives (Lubberink, 2020). This phenomenon is referred to as mission drift (Fowler, 2000; Weisbrod, 2004; Jones, 2007; Ebrahim et al., 2014). As social enterprises are set out to achieve *both* social and financial performances, compromising one of their values leads to a change in the identity and nature of the enterprise or to tensions at least (Smith et al., 2013).

### Personal Motivations for Social Entrepreneurship: The Role of Empathy

Traditionally, financial gain, creative freedom, and control over one’s own efforts are seen as motivations for entrepreneurship. Social entrepreneurship requires additional motivations, and balances self-interest (financial gain) with the interest of the beneficiaries of the enterprise (gain of others). Miller et al. (2012) show that social entrepreneurs are motivated by both rational, self-focused factors (self-realization, autonomy, and performance behavior), and emotional drivers, such as compassion. On top of these self-focused motivations, they are driven by contributing to the well-being of others. In a comparison between social and commercial entrepreneurs, Clark et al. (2018) show that social entrepreneurs often set more ambitious goals than their commercial peers. Also, the level of entrepreneurial self-efficacy is higher for social entrepreneurs than for commercial entrepreneurs.

Several studies have investigated personality traits of social entrepreneurs (e.g., Rauch and Frese, 2007; Koe Hwee Nga and Shamuganathan, 2010; Obschonka and Stuetzer, 2017). In general, these studies point toward a “prosocial personality” that distinguishes social entrepreneurs from traditional entrepreneurs. Prosocial personality is defined as “an enduring tendency to think about the welfare and rights of other people, to feel concern and empathy for them, and to act in a way that benefits them” (Penner and Finkelstein, 1998, p. 526). Studies about intentions to engage in social entrepreneurship indicate “a sense of moral obligation” as an additional personality trait of social entrepreneurs. Moral obligation refers to the feeling that one is obliged to do something about someone else’s needs. This moral obligation comes from within a person, but can also be the result of, for example, social pressure or a certain social standard (Hockerts, 2017; Stephan and Drencheva, 2017; Ploum et al., 2019).

Empathy as a concept is characterized by a broad array of definitions, which vary from reflecting a narrow, well-defined perspective, such as the one provided by Coplan (2011, p. 40): “a complex, imaginative process through which an observer simulates another person’s situated psychological states while maintaining clear self–other differentiation;” to a broad, all-compassing one, e.g., as presented by Cohen and Strayer (1996, p. 988): “understanding and sharing in the other’s emotional state or context.” The role of perspective-taking is central to understanding “pure” empathy (Coplan, 2011). Empathy requires other-oriented perspective taking, i.e., to recognize other people’s emotions, while being aware that these emotions are not one’s own and preserving a clear distinction between self and other (Carré et al., 2013). Empathy, sympathy, and compassion are often used interchangeably in the literature, yet there are conceptual differences (Carré et al., 2013; Jeffrey, 2016). For example, Jamison (2014) indicates that empathy, unlike compassion or sympathy, is a deliberate choice to adopt another person’s perspective and imagine how one would feel, think, and desire if one were in the other person’s position (Coplan, 2011). In contrast, sympathy and compassion are reactive responses rooted in concern for the welfare of others (Jeffrey, 2016), which are automatically evoked when something bad happens to another person. Moreover, sympathy consists of feeling an emotion for the other person (self-oriented) – for instance, imagining how one would feel if one were in the other person’s position – rather than feeling an emotion from the perspective of the other person (other-oriented) – for example, imagining how the other feels while being in their position (Eisenberg, 2010).

Over the years, empathy has been understood as multidimensional concept, including cognitive and emotional components (Davis, 2006, 2015). It has been argued that empathy contains different (interlinked and interacting) components, including an affective (emotional), a cognitive, a behavioral, and a moral component (Morse et al., 1992; Jeffrey, 2016). Affective (emotional) empathy refers to “the ability to subjectively experience and share in another’s psychological state, emotions or intrinsic feelings” (Morse et al., 1992, p. 274). You feel the emotional state of another person as your own (Morse et al., 1992; Jolliffe and Farrington, 2006; Wisnewski, 2015; Jeffrey, 2016). Cognitive empathy refers to the ability to take an objective stance toward the emotional state of another person, i.e., being able to cognitively understand the other person’s emotion (Morse et al., 1992; Jeffrey, 2016). Behavioral empathy denotes a communicative aspect of empathy. One conveys to the other person that one understands the other’s perspective (Morse et al., 1992; Irving and Dickson, 2004). Finally, moral empathy refers to a concern for the other and the desire to relieve suffering, which is driven by altruism. Moral empathy fuels the motivation to act prosocially and altruistically (Batson, 1991; Decety, 2015; Jeffrey, 2016; Melchers et al., 2016; Surguladze and Bergen-Cico, 2020). Quantitative research of Bacq and Alt (2018) supports the absence of a direct link between empathy and social entrepreneurial intentions. Empathy is channeled by two mediating mechanisms, i.e., social entrepreneurial efficacy and social worth.

In the remainder of this article, the terms “moral empathy” and “empathy” are used interchangeably to refer to moral empathy. Empathy is also referred to as a trait, referring to its relatively stable character in contradiction to situational empathy that can vary according to the situation (Bacq and Alt, 2018). Survey-based studies measuring empathy have frequently used the Basic Empathy Scale (BES; Jolliffe and Farrington, 2006). This scale contains a dual-component of empathy, including items that reflect affective and cognitive empathy. The validity and predictive value of the BES is confirmed in various studies, yet this scale has limitations as well. As later studies have distinguished more dimensions of empathy, several revisions have been made to the original BES. A prominent revision by Carré et al. (2013) includes three components of empathy, i.e., emotional contagion, emotional disconnection, and cognitive empathy. Interpreting data on the basis of these scales is difficult, because it is hard to disentangle self-oriented perspective taking from other oriented perspective taking.

#### Empathy and the Theory of Universal Values

This study focuses on the personal motivations of social entrepreneurs and contextual factors that influence their motivations. A theoretical basis is drawn on the framework of Universal Human Values (UHVs; Schwartz, 1994), as it is often used as a tool for research into the motives of social entrepreneurs (Stephan and Drencheva, 2017). The theory of UHVs identifies 10 basic types of values, namely Universalism, Benevolence, Tradition, Conformity, Security, Power, Achievement, Hedonism, Stimulation, and Self-direction. Later, these were further refined in order to better represent the continuum of related values (Schwartz et al., 2012). It is likely that a social entrepreneur will be driven by strong “self-transcendent” values (Universalism and Benevolence), which emphasize enhancement of others and transcendence of selfish interests. Interestingly, the theory of UHVs conceives Benevolence (empathy) to be directly opposed to Achievement (performance), highlighting the apparent contradiction between these values (Balliet et al., 2008). Similarly, in the context of sustainable entrepreneurship, Ploum et al. (2019) accentuate the contrast between the “self-transcendent” values and the “self-enhancement” values.

To bridge this gap between opposing values, social entrepreneurs may resort to finding moral justifications for their commercial activities (Ebrahim et al., 2014; Ploum et al., 2019). Yet, this harbors the risk of losing sight of the social outcomes of commercial activities, i.e., means-ends decoupling (Bromley and Powell, 2012). This study refers to the framework of UHVs by recognizing, classifying, and coding the values exposed by social entrepreneurs. Specifically, Benevolence (empathy) is studied as a driver for founders of social enterprises. Several studies have associated empathy with having a prosocial personality, which stimulates individuals to pursue social entrepreneurship (e.g., Batson, 1991; Koe Hwee Nga and Shamuganathan, 2010; Miller et al., 2012; Ruskin et al., 2016; Waddock and Steckler, 2016).

#### Triggering Empathy by a Critical Life Event

Personal context and experience play an important role in the motivation to start a social enterprise (Stephan and Drencheva, 2017; Saebi et al., 2018). Personal experiences can take many forms. Entrepreneurs may experience shortage of social provision, may be involved in a traumatic event, may endure poverty during their childhood, or may be initiated into a family tradition of volunteering (Stephan and Drencheva, 2017). These can be described as transformative early adulthood experiences (Shumate et al., 2014) that may lead to social engagement. Entrepreneurs who experienced a problematic situation in their youth may want to address a certain issue now that they are adults. Others see current social needs unfulfilled and focus on resolving it in an entrepreneurial way.

Social entrepreneurs often share their background with the population they wish to serve with their business (Saebi et al., 2018). Role models, family traditions, and tax incentives can also play a role in the development of prosocial entrepreneurial behavior (Stephan and Drencheva, 2017). These personal experiences appear to strengthen feelings of empathy, and as a result, these individuals are more likely to start a social enterprise (Yiu et al., 2015). In an investigation of life events, Yitshaki and Kropp (2016) distinguish three types of motivations for starting a social enterprise: (1) finding solutions to unmet social needs based on past and present experiences (pull factors, e.g., coping with a problem from the past); (2) internal motivations based on identification with social needs and process evolution of an idea (pull factors, e.g., influence of parents who were socially aware); and (3) identification with social needs or process evolution of an idea (push factors, e.g., searching for a meaningful career). As personal experiences can induce feelings of empathy, empathy is identified as an important mechanism *via* which the start of a social enterprise is triggered (Yitshaki and Kropp, 2016).

## Materials and Methods

### Method and Data Collection

Despite the recommendation to increase quantitative research on social entrepreneurial personality (Stephan and Drencheva, 2017), this study adopts a qualitative research design. Both approaches are complementary and have a different purpose. Qualitative research is chosen here for understanding the role of empathy and critical life events as triggers for social entrepreneurship. This particular design is relevant when the aim is to gain insights into “how” and “why” questions. This approach is particularly appropriate when not all variables have yet been mapped and there is a need for an in-depth investigation of a phenomenon in its natural context (Saunders et al., 2009; Thomas and Magilvy, 2011; Hayashi et al., 2019). As the goal of this study is to unravel the motivations and values of social entrepreneurs, an exploratory design using semi-structured interviews was set-up. Asking open questions and follow-up questions uncovers new in-depth information (Saunders et al., 2009). Instead of focusing on single case studies, there is a need for systematic case comparison based on a large-scale database (Dacin et al., 2011). This research responds to this urge by systematically analyzing semi-structured interviews of 33 social entrepreneurs.

The sample consists of Belgian social entrepreneurs, located in two regions of Belgium: Flanders and Brussels. One of the difficulties with respect to research on social entrepreneurship is the lack of a clear definition and the absence of a list of social enterprises. Following Dacin et al. (2011, p. 1204), the social mission is the most important distinguishing element, i.e., “creating social value by providing solutions to social problems.” Others (e.g., Portales, 2019) call it the *raison d’être* of social enterprises. The selection took place by “convenience sampling,” and entrepreneurs were selected on the basis of two criteria: (1) the organization has a prominent social mission and (2) the organization combines its social mission with an economic activity. For this study, founders, directors, and executives of social enterprise organizations were interviewed. The interviews took place in 2018 and 2019, within the frameworks of two university courses on social entrepreneurship, coordinated by two of the authors. For reasons of privacy of the interviewees and possible sensitivity of company information, the organizations are anonymized and numbered. To get a sense of the organizations, Table 1 provides an overview of the type of the social enterprise, i.e., its main focus or activity and legal status.

**Table 1:** Details of the social entrepreneurs and organizations in the sample.

Attached number case	Main focus or activity	Legal status
1	Providing alternative programs for students (at risk) of dropping out of school	Not-for-profit association
2	Supporting people with a distance to the labor market	Not-for-profit association
3	Supporting social vulnerable people in their search for work	Not-for-profit association
4	Supporting people with a distance to the labor market	Not-for-profit association
5	Fighting energy poverty	Social Cooperative
6	Supporting people with a distance to the labor market	Not-for-profit association
7	Providing energy loans for socially vulnerable people	Social Cooperative
8	Supporting people with a distance to the labor market	Not-for-profit association
9	Travel agency for people with disabilities	Private company
10	Renovating homes of socially vulnerable people	Cooperative
11	Supporting people with a distance to the labor market	Not-for-profit association
12	Supporting people with a distance to the labor market	Social cooperative
13	Daycare for people with disabilities	Not-for-profit association
14	Supporting people with a distance to the labor market	Not-for-profit association
15	Supporting people with a distance to the labor market	Not-for-profit association
16	Travel agency for people with disabilities	Not-for-profit association
17	Daycare and home for people with autism	Not-for-profit association
18	Supporting sustainable energy	Cooperative
19	Daycare for people with disabilities	Not-for-profit association
20	Childcare	Not-for-profit association
21	Daycare and home for people with disabilities	Not-for-profit association
22	Providing training for disadvantaged young people	Not-for-profit association
23	Supporting people with a distance to the labor market	Not-for-profit association
24	Supporting local and sustainable production and consumption	Private company
25	Renovating buildings for social purposes	Social cooperative
26	Investing in renewable energy	Social cooperative
27	Supporting people with a distance to the labor market	Not-for-profit association
28	Fight poverty by offering work for the less fortunate	Not-for-profit association
29	Online web-shop for handmade products	Private company
30	Thrift shop, wood, and bike workshop supporting social vulnerable people	Not-for-profit association
31	Developing creative and sustainable answers to (main) urban problems of society	Not-for-profit association
32	Providing flats and houses at a reasonable price	Not-for-profit association
33	Supporting people with a distance to the labor market	Not-for-profit association

### Rigor of the Research

One of the peculiarities of qualitative research is the different way of establishing trust in the findings of the research. Rigor of this study is dealt with in line with Morse (2015) who argues that the same terminology has to be used consistent with quantitative research, i.e., validity and reliability, but that the strategies differ. Morse (2015) is critical about the common strategies in qualitative research and argues that still a lot of work needs to be done to establish rigor. Daniel (2019) gives indicators in an effort to counter the contemporary polarization regarding rigor in qualitative research. Thomas and Magilvy (2011) also propose a model to enhance trustworthiness. These three contributions are combined to explain the rigor of this qualitative research.

Strategies for external validity, or generalizability, focus on transferability. The findings of this study can be applied to other settings. The 33 Belgian social enterprises provide homogeneity of the sample (e.g., regarding geographical context, culture, and legislative context) and reassurance of the inter-case comparison. The findings also have meaning for international contexts. Similar to other studies, like Shumate et al. (2014), reporting on the influence of family legacy of 20 social entrepreneurs based in the United States (with the exception of one which was based in Malaysia), and Kimmit and Muñoz (2018) analyzing sense making processes of 15 Chilean social entrepreneurs, lessons can be learnt from the role of moral empathy as a trigger for social entrepreneurship.

Strategies for internal validity stress the credibility of the research. This study is optimized by employing an extensive literature research and by interpreting the concepts and constructs based on the existing literature. For each interview, the same questionnaire was used. Questions were carefully formulated in an open way. This procedure enabled the researchers to receive a truthful picture of the motivations of the social entrepreneur, without leading respondents into certain directions. The questionnaire had no explicit questions probing empathy or life events, so steering answers was avoided. Interviews were recorded and transcribed before connections between concepts were made and conclusions were drawn. The conduction and the analysis of the interviews were done by different researchers, thereby reducing the risk of biased interpretations.

Interviews were transcribed and coded, using Microsoft Excel. First, key aspects were identified by labeling the utterances of each individual participant on motivations, personality factors, and life events. Initially, open codes were used to identify categories. The literature was used to increase theoretical understanding, and concepts and constructs from theory were used to categorize and code the answers of respondents. Codes for empathy were inspired by the literature on different components of empathy, as well as the BES (Jolliffe and Farrington, 2006). These codes were helpful as a guideline to signal the possibility that a statement was related to empathy. Personality traits were coded based on the Big Five personality traits (Goldberg, 1990). Codes for values of social entrepreneurs were derived from the theory of UHVs (Schwartz, 1994). For example, the concept “altruism” is not easily mentioned by the interviewees, but due to the theory of UHVs links could be made to underlying categories, such as “meaning of life,” “true friendship,” and “helpfulness.” In this way, the codes could be connected to the theory, which contributed to constructing validity. Second, labels were grouped and when possible categorized or merged (axial coding). Finally, data were grouped into higher-order themes, and connections were made between themes. Table 2 shows the code tree. In addition to empathy, several other motivations appeared to be important to the interviewees, such as self-realization and meaning. These motivations were also included in the code tree.

**Table 2:** Codes and constructs.

Common descriptions	Code	Theoretical construct
Feeling, empathy, and compassion	Empathy	Empathy; sympathy; and compassion
Upbringing, disappointment in the business world, and signaling “gap in the market,” personal experience	Personal experience	Life event
Autonomy, self-management, personal freedom, status, influence, and job satisfaction	Self-realization	Self-direction/performance drive (UHV)
Sense/satisfaction/impact	Meaning	Meaning in Life (in Altruism, UHV)
Intrinsic motivation/drive/passion	Drive	Personality traits (Big Five) Achievement (UHV)
Social commitment, giving something back, adding value for others (self-sacrifice/altruism/no status/social justice), being able to figure it out, not working for the money, identifying social problems	Social commitment	Altruism and Universalism (UHV); Altruism (Big Five)
Perseverance/resilience	Continue	Personality traits (Big Five)
Challenge/performance/entrepreneurship/risk/innovation/growth	Entrepreneurship	Performance drive/Self-direction (UHV)

Triangulation was used to establish validity. Conforming other qualitative studies, different data analysis techniques (e.g., building timelines, codification, and case comparison) are combined to unpack the motivational and contextual factors that influence social entrepreneurs. Interview questions were anchored in the literature and related to motivations, personality traits, and contextual events of social entrepreneurs. Key questions were: “What was the reason for founding the company?;” “What motivates you to work?;” and “What characteristics, knowledge and qualities do you possess that help you to be a social entrepreneur?” Furthermore, secondary data were collected from sources, including corporate websites, presentations, and news articles. These sources were suitable to verify events and the social and economic value creation of the social enterprises.

Strategies for reliability highlight the dependability of the study. Before the start of the interview, interviewees were asked for consent to use the interview for research purposes, as well as permission to record the interview. One of the respondents only gave permission to make use of the data. In this particular case, the handwritten notes were analyzed. All interviews were transcribed, and upon request the resulting transcriptions were sent to the respondents to check for inconsistencies. The transcriptions were coded by one of the researchers and independently checked by two other researchers, after which results were compared and discussed.

## Results

### Empathy

In order to recognize (different components of) empathy in the interview transcripts, coding was inspired by the literature on empathy, such as items from the BES (Jolliffe and Farrington, 2006), as well as items related to Benevolence (empathy) in the framework of UHVs (Schwartz, 1994; Schwartz et al., 2012). From the interview texts, different components of empathy could be identified, indicating that the “feeling” and the “understanding” often go hand in hand. The following sections will further explore how moral empathy played a role in entrepreneurial activities, as social entrepreneurs are dealing with solving specific (social) problems for vulnerable target groups. It was found that when empathy played a role, it was a motivator for prosocial behavior. Empathy played an important role for the interviewees, because the concept was often raised, explicitly or implicitly, without the questions focusing on it.

Empathy results from a special involvement with a certain target group. Some respondents have a special sensitivity or connection with a target group and, therefore, like to take action when they are moved by something. The following quote is an example of how moral empathy plays an important role in the actions of the social entrepreneur:

For me this is the fundamental commitment to vulnerable people in this society and that is mainly homeless people, people with a social disadvantage, with an alcohol problem, psychosocial disadvantage, drug problem, divorce etc. This is a large group that was underprivileged then and unfortunately still is (#8).

These entrepreneurs already have an empathic connection. They see a problem or an opportunity and go for it. There is clearly an empathic attitude and the interviewees also mention it as an important requirement to work in the organization.

If you (…) are someone who communicates badly or does not ‘feel’ at all, you cannot become a good social entrepreneur. Actually it starts with a strong motivation of course. So, you need to have a drive, you have to want to realize something. (…) But most of all … yes, the heart in the right place. Not the social seeing of “oh that’s a hype, that’s a good way to make my company a profitable business”. I do not think that’s the right attitude either. It’s actually a mix of wanting to do business but with a social sensitive… with a social commitment (#6).

A “social character” (implicitly) is seen as a feature that a social entrepreneur should have. As the following quote shows, social commitment and taking action in order to help socially vulnerable people are examples of moral empathy. This respondent also points toward links and differences between social and “commercial” entrepreneurship.

The great thing about social entrepreneurship is that you can link ideals and social commitment to very concrete plans. You do not just talk about the things you think are important, you actually do something, you make a difference. When you are in the social economy, that underlying commitment always comes to the surface, but you also have to try to learn from the traditional entrepreneurs. They work on the basis of the classic pursuit of profit, but they can also have a social character. For example, the fact that he can employ people, but social entrepreneurs are still different (#32).

These respondents mention what in their eyes distinguishes the social entrepreneur from their commercial opponents. Other interviewees do not always use so many words, but show with their answers that they empathize with the people they are trying to help.

The smile of a person who has been helped or you see someone who lived on the street who proudly comes to tell after three years: “I have a wife, my first baby was born. We live in a small one bedroom apartment”, but he is still happy that he is making something of his life (#31).

The quotes indicate that empathy is addressed in the interviews in various ways. However, not every respondent talked about empathy. For some, this can be explained by the nature of the organization and the scope of the social impact envisioned. For example, a sustainable energy cooperative or a bicycle shop might differ from not-for-profit organizations that aim to provide daycare for vulnerable people, and might adopt some kind of economic activity to cover their costs. Some of the entrepreneurs with a social objective and target group do not explicitly voice the empathic side of their activities. These respondents talk mainly about administrative matters. For others, there seems to be a total lack of empathy as a motivation for their activities. They do not even talk about social impact, but solely stress the financial bottom line or the variety in their work. When asking what motivates them to go to work, the following aspects come to the fore:

But we actually have to manage this like an economic organization (…) Of course we must also ensure that we make profit (#2).

It’s a challenging job and the job is also a diverse one. My job is in- and outdoors. It can be about the administration, the staff, making quotations, making the designs or having conversations with clients. It is a very varied job (#12).

The respondents mainly talk about being able to realize one’s own needs and wishes, and little or no mention is made of the social impact of their work, or the target group for which the work is being done. The degree of empathy of social entrepreneurs in the sample of this study is diverse. For some, empathic feelings are the main reason for their work, while for others this is less important. For a number of interviewees, the social goal does not even seem to play a role at all. These findings provide a nuanced picture in response to the question which role empathy plays in the motivation of social entrepreneurs. Empathy turns out to be a driver for a considerable number of entrepreneurs, but not for all of them. Sometimes, the empathic attitude of the entrepreneur has a clear origin or reason, which will be further explored in section The Role of Life Events.

### The Role of Life Events

Personal circumstances or “life events” can have been catalysts that brought the interviewee to their current professional position. In some cases, the life event concerned the founder of the organization, and in other cases, the life event was experienced by someone with a managerial position within an existing social enterprise. All interviews that discuss life events are described according to the themes distinguished by Yitshaki and Kropp (2016) with the exception of “social actions as a spiritual necessity,” as this theme was not detected in the interviews. Also the group “social awareness since childhood or early adulthood” is not included in the results section. Although this theme was detected in one of the interviews, it could not be included in the results section because the respondent could become indirectly identifiable.

The quoted “life events” or transformative experiences are not always the stories of the interviewees themselves. They can also be “corporate origin stories,” i.e., a story, romanticized or otherwise, about the founding of the organization, before the interviewee started being employed at this organization. Not all “life events” have led to the establishment of the organization in question. In a number of cases, it is a “life event” that leads to a different career path, as a result of which the interviewee made the switch from commercial enterprise to the social organization. On the one hand, a life event might lead to the establishment of the organization, and on the other hand to a career switch. Yitshaki and Kropp (2016) qualify the life events that led to the creation of a company as “pull factors,” while the “push factors” led to a career switch. The literature review assumes that a “life event” has a positive influence on the degree of empathy, as a result of which a social enterprise is then started for the target group to which the empathy emanates. The interview data show that a “life event” has influenced the career or life path of a large number of interviewees to such an extent that they now work for a social enterprise. As such, transformative experiences such as personal circumstances or life events can create more empathic involvement and bring entrepreneurs on the path of social entrepreneurship.

#### Solving Unmet Social Needs Based on a Current Problem

Several entrepreneurs started their social enterprise when they identified an unfulfilled need with a (socially vulnerable) target group. These entrepreneurs all discovered a gap in the current offer for their target group. A personal problem was the reason to set-up an organization that would tackle this problem for other people in similar situations. Because they were touched by what they saw, heard and experienced on a personal level, they decided to set-up an entrepreneurial initiative to help the target group. The following quotes illustrate this:

When we got there, they had just received the news that one of their biggest funders had said “from next year you will not get any more funding from us”. (…) And then we were thinking about how we could actually bring a little bit of money into the picture. We just wanted to make sure that the NGO, instead of having to rely on funding for a while, could actually generate its own money through something they produce (#29).

The organization was founded years ago (…) They were a group of friends, who had problems with the AIDS patients who were dying in the streets at the time. And actually that’s where [organization] started (#31).

Then I had an accident. (…) At that moment you end up in a totally different situation. (…) I wanted to do something with all the knowledge I gained (…) for other people. Also because I could not find a company that did this, I decided to start an organization myself (#9).

#### Solving Unfulfilled Social Needs Based on Past Experiences

Turning a (negative) experience of the past into positive action for the future, that is what happened at one of the companies, as shown in the quote below. Entrepreneurs may have encountered certain negative experiences in their past, such as being involved in a violent life event (mentioned by interviewee 22), or being confronted with poverty (mentioned by another interviewee). In this context, Yitshaki and Kropp (2016) refer to “personal rehabilitation for past events.” The motivation for becoming a social entrepreneur may then be rooted in a certain sense of empathic understanding of the social problems of others.

(…) was mugged (…) years ago by a number of young people. (…) He was thinking about that when he was in hospital. And his reaction to that was actually that these young people do have talents and work well together but use them totally wrong (#22).

#### Ideological Motivation

One of the interviewees does not clearly describe a life event, but instead mentions an ideological driver. One of the entrepreneurs saw an opportunity to make housing more social, which fitted well with his ideological direction. The interviews did not always explicitly state ideological motivations, but between the lines several ideological points of departure could be identified. After all, every entrepreneur has his or her own views on life and an image of what the world should be like. The following quote represents an ideological driver:

For me, social innovation must really lead to change. I’m quite anti-capitalist about ideology, I mean the invisible ideology which is something else than always the desire for more profit, more growth. I would not say it’s the intention, for example, to destroy the classic model (…), but I strongly believe in the importance of an ecology of different kinds of views on what an economic system is (#32).

#### Natural Option for Career Development

Some interviewees mentioned dissatisfaction with their previous job. The search for a meaningful job and wanting to change a career path can be reasons to start with a social enterprise, or switch to an existing social enterprise. For these interviewees, the move to the social enterprise was attractive because of the dissatisfaction and negative experiences with previous jobs and commercial companies.

So, I was a HR manager in a badly running company. And when you saw how people were treated in this company, I did not see myself doing this all my life (#2).

I used to work in the private sector. That company had gone bankrupt and I was looking for something else. I deliberately made the switch to the social sector. My previous company, that was a private company, and I was in [abroad] a lot. And by working with target groups there, I wanted to make the switch (#13).

In several interviews, the interviewees indicated that at a certain moment in their life they experienced a flash of insight, for example, when retirement was approaching. Or, after a few years in the private sector, interviewees began to wonder whether they wanted to do this job for the rest of their life. For some social entrepreneurs, the transition to a social enterprise was made because they reached a certain age and had the feeling that life is short.

I have come at an age when you are actually starting to think about (…), eh when you are allowed to retire, so um what can I say? I’ve always been very social and I actually wanted to give back a bit for society, (…) a lot less than what you can earn in private but at some point in your life you need that less. When you are young, you have to buy a house, a nice car, so then you need more money. Now you can say “I want to give something back to society” (#31).

I found that fascinating but after the third year I began to wonder what I want to do until the end of my life. Then I did not think it would add any value and that I wanted to do more in my life than just that (#28).

Before I came to work here, I worked in the regular economy for years, within six companies of which I worked as a commercial director (…), and what struck me there was a very strong focus on the profit. This is very nice if one achieves this of course (…) Actually, apart from the bonus that came on your account, for the rest it was not motivating to do this (#4).

The interviewees indicated that their work and life experiences up to a certain moment in time made them understand that they wanted to do something that had a clear social value for others.

Various interviewees reflected on their extensive life experiences before starting a social enterprise. However, among the interviewees were also young people who, sometimes during or immediately after their studies, started a social enterprise. There seems to be no direct link between age and the decision to start a social enterprise. Yet, the interviews show that students and recent graduates often set-up their own social enterprise (e.g., based on ideological principles), while more experienced entrepreneurs tend to start working in an existing social enterprise, mainly because they want to change their career path.

We have now decided, we do not want to earn anything so all the profit we make in the future, is actually a buffer to further expand and to continue to set up projects. That’s real, we first want to grow our business and grow until we earn something ourselves, so that’s it (#24).

### Other Triggers and Motivators

#### Self-Realization as a Motivator

Next to empathy and life events, other factors motivate and influence the social entrepreneurs. Autonomy, pride, personal freedom (belonging to the value Self-direction), and influence (forming part of the value Achievement), all turn out to be important motivations for the interviewees. These concepts have been coded and combined as “Self-realization.” Self-realization was important for those entrepreneurs who referred to empathy and also for those who did not. Self-realization emerged as a motivator, without being specifically asked for during the interviews.

What gives me strength is that you can keep renewing the organization, and that you can keep renewing an organization that also remains inspiring … sometimes a bit provocative … that is inspiring for the environment around us. To offer things from: “that’s the way to do it” and “that’s the way it can go”. That way you can approach a number of social issues. For me, that’s actually the motivation to draw a line there that would not otherwise be drawn (#33).

While I think I have a good salary, but that’s also important to me, you also have to pay bills, but because of the fact that the social part is there, you also see that if I go somewhere in a restaurant and I see someone there who we have trained then I am super satisfied (#28).

I think what I like most about myself is that I think our story is right. That I do not do production or do work that I would not want to do myself, in the sense that, if you look around, I can find goods here every day that I say: “wow, handsome and clean, does that end up in that dump?” Actually, I think the most, even the most satisfying thing is that we can create jobs by selling reusable goods. We can actually be creative, we can have fun, we can have fun. Um, that’s the fun part (#15).

That’s actually an intrinsic motivation. I was able to develop it myself, develop all my ideas (#2).

#### Social Engagement

A motivation that the interviewees regularly mentioned is “social engagement.” For example, they mentioned “added value for society,” “giving something back to society,” and similar terms. Social engagement does not seem to be the same as moral empathy in the interviews, although both are clearly related to the “self-transcendent values.” Wanting to do something in return for society, not just making a profit and adding value for others, is apparently a different driver than empathy and compassion. The socially engaged entrepreneurs also do not always mention empathy as a driver, and the empathic entrepreneurs do not always mention social engagement as a motivating factor.

When I’m seen like that, that sounds great of course, yes of course because I do not want to do business and just sell glue for example and make money with it, no I really want to and I’ve made that clear several times in the meantime I think, I really do want to do business to create a social impact actually (#24).

By my nature, I could not be any different. I’m not anyone’s rock-solid financier. Social entrepreneurship also means that your profit is not an end in itself. Your profits go to your social entrepreneurship. It has to be added value for society, that’s the most important thing for me. To be able to mean something for your environment, for your fellow human beings, yes that’s very important to me (#20).

#### Meaning and Satisfaction as Motivation

Working for a social purpose gives meaning and satisfaction. Sometimes, the interviewees contrast the satisfaction they perceive from working for a social purpose with the satisfaction they experienced in previous jobs. The meaningfulness and satisfaction that the social entrepreneurial work offers turn out to be important “drivers” for the entrepreneurs to (continue to) do their work.

Above all, therefore, the social objectives. The motive to help people may sound a little too soft, but it’s mainly about things that matter, that are useful instead of pursuing pure profit and getting rich and going more for human achievements (#3).

The difference here is that I can really mean something in the employment of those people. What I personally experience is that if one can be useful and start working with a good feeling that the added value that one can have to society is simply higher (#4).

The challenge, but also the feeling of being here is not only for myself but what I do also makes sense for others. And that has to do with meaning (#6).

#### Drive

The word “drive” recurs regularly in the interviews, without explicitly being asked for. Although each interviewee mentions drive in a different sense, it seems to be a character trait that is key to social entrepreneurship. Drive seems to be synonym with intrinsic motivation, tenacity, and sometimes passion. Drive is seen as an inner motivator to get something done. Furthermore, the quotes also point toward the connection between drive and empathy, as they mention taking care of employees or target groups, and being willing to take leadership in difficult positions.

That social piece always gives that extra drive. (…) A gigantic drive against the current. And it’s really against the current, because everyone is going to say it’s not feasible. For me it is very clear: social entrepreneurship is a passion, with a lot of drive, entrepreneurship in a way I believe it works, but with my feet on the ground (#1).

You need to have some kind of innate drive to take on leadership. Because entrepreneurship is first and foremost taking leadership (#5).

So you must have a drive, you must want to realize something (…) You have to keep feeling that drive. (…) If you do not have that drive anymore, you have to, you have to stop, you have to do another job (#6).

I think what is positive about me is that I still have a huge drive and that I am indeed going for it. That I stand behind my staff. I cannot deal with it when people are negative about my staff in a certain place, then I react and I jump in the breach, they know that about me too (#8).

## Discussion

### Empathy as a Driver for Social Entrepreneurs

In several previous studies, empathy is seen as the most important distinguishing characteristic of a social entrepreneur (Miller et al., 2012; Bargsted et al., 2013; Ruskin et al., 2016; Lubberink, 2020). This study does not make a comparison between “traditional” entrepreneurs and social entrepreneurs, but looks at the role of empathy in the motivation of a social entrepreneur. As described in the results, empathy is an important driver for social entrepreneurs, but not for all of them. Other, more mundane motivations also appear to be important. These include, among others, self-realization and drive, motivators and triggers that are associated with “traditional” entrepreneurs as well. These results confirm earlier research concerning empathy and social entrepreneurship in two ways. First, it provides qualitative evidence of the link between empathy and social entrepreneurship that has been made (quantitatively) in previous studies (e.g., Bacq and Alt, 2018; Wu et al., 2020). Second, it provides the view of experienced social entrepreneurs in addition to studies that are based on nascent perspectives by means of student questionnaires (e.g., Bacq and Alt, 2018; Ploum et al., 2019; Wu et al., 2020).

Stephan and Drencheva (2017) already indicated that a social entrepreneur does not differ that much from the “ordinary” entrepreneur on several points. Like a commercial entrepreneur, the social entrepreneur is driven by values, such as independence, performance, and influence. Also Clark et al. (2018) found that a social entrepreneur differs on some points, such as a higher level of ambition and a higher assessment of his or her own abilities. On other points, however, the differences are less pronounced, for example on the point of “self-centeredness.” This is confirmed by the results of this study. Important drivers for entrepreneurs, such as autonomy, freedom, and the ability to develop independently (all encoded under “self-realization”) were found among almost all the interviewed social entrepreneurs. What might be specific for social entrepreneurs – exceptions aside – is that they mentioned social commitment and being proud to realize something for others. This is also in line with the findings of Bacq and Alt (2018), who found that empathy indirectly affects social entrepreneurial intentions among students.

In previous studies, empathy (sometimes labeled as “compassion”) is seen as the distinguishing factor for social entrepreneurs, among others by Miller et al. (2012). The fact that some entrepreneurs show empathy is clearly reflected in the results. At the same time, a substantial proportion of the entrepreneurs was not driven by an empathic attitude. These entrepreneurs set more focus on economic value creation of their organization and, thereby, tend to have drifted away from the social mission of their organization. By emphasizing the economic impact of their organization, they drift toward a profit focused business model characterized by financial impact first. This phenomenon refers to growing commercialization (e.g., as a result of diminishing funding or neoliberal influence) and is in line with previous research focusing on growing tendencies to stress economic outcomes rather than social ones (e.g., Comforth, 2014; Maier et al., 2016). Questions arise regarding the role of empathy in this process: Was mission drift the result of an overall lack of empathy; or were these social entrepreneurs empathic when starting their venture and somehow lost or neglected their empathic drivers while running their organization? In such particular context, one can relate this type of empathy more with situational empathy (Bacq and Alt, 2018).

In any case, the differences between these types of social entrepreneur – lacking empathy from the start or losing it along the road – and their commercial counterparts is becoming small. Furthermore, it shows that prevailing focus on the social dimension is needed, in order to prevent mission drift. This is in line with Wu et al. (2020) who found high levels of empathic concern among nascent social entrepreneurs (business students) to be an important factor as opposed to moral disengagement and elements of the dark triad, i.e., narcissism, psychopathy, and Machiavellianism. It would be interesting to explore these issues of mission drift in relation to empathy and the differences between social and commercial entrepreneurship in more detail in future research, as well as its link with business education with focus on social and sustainable entrepreneurship (e.g., Lambrechts et al., 2018). Specific links with dark triad aspects might also lead to future research in which empathy is further analyzed from a cognitive neuroscience perspective (Decety, 2015; Surguladze and Bergen-Cico, 2020).

In the theory of UHVs (Schwartz, 1994; Schwartz et al., 2012), opposing values are assumed to conflict. In this research, this would mean that prosocial motivations such as empathy or social engagement (on the side of Universalism and Benevolence) conflict with self-realization and “drive” (Achievement and Self-direction). While Sastre-Castillo et al. (2015) found values related to self-transcendence and conservation to be important factors influencing social entrepreneurs, there seems to be no internal conflict among the entrepreneurs in the sample of this study: Many of the empathetically driven social entrepreneurs indicate that they are also motivated by self-realization. The drive for self-realization is in line with a comparative study by Clark et al. (2018), who found that social entrepreneurs often set higher goals and rate their own ability to achieve them higher than the commercial entrepreneur. Whether social entrepreneurs experience a conflict between these values and the way how they deal with such conflicting values is an interesting topic for future research.

The literature on empathy assumes that women have a higher degree of empathy than men (e.g., Jolliffe and Farrington, 2006; Carré et al., 2013). Such gender preferences are confirmed in this qualitative study, as the interviewed women are more often motivated by empathy than men (eight women vs. four men), or at least mention this (explicitly or implicitly) during the interviews. The fact that (male) entrepreneurs are not talking about the “soft” side of social or sustainable entrepreneurship, such as normative elements, or in case of this study, empathy, might be in line with previous research in the context of sustainable entrepreneurs, that were found to be hesitant to present themselves as being ethical or behaving according to normative competences. They rather preferred to present themselves as taking strategic or future-oriented business decisions (e.g., Lambrechts et al., 2019). This positioning of entrepreneurs as being empathic or normative, as well as the gender preferences displayed in this research, provide interesting research avenues to be further explored.

### Life Events as a “Trigger” for Social Entrepreneurship

According to previous studies, experiencing a “life event” can influence the life course and choices of social entrepreneurs (Shumate et al., 2014; Stephan and Drencheva, 2017; Saebi et al., 2018). In this study, results show that a life event has greatly influenced the life path of some interviewees. The life event sometimes led to the start of the company, or to a career switch. Yiu et al. (2015) found that a life event could strengthen the feelings of empathy, increasing the likelihood of someone starting a social enterprise. In this study, that pattern (life event leads to stronger feelings, which leads to starting a business) is clearly recognizable in some entrepreneurs. There are indications that a life event can be a landmark for doing something for a certain target group. Some examples have already been given, such as experiencing that there is no support for certain socially vulnerable groups or being personally affected by disaster. These life events have a great deal of influence on the further course of the entrepreneur’s decisions.

Among others, Yitshaki and Kropp (2016) write about “search for meaningful career” as a motivating factor for social entrepreneurs. Meaning is not included as a separate motivator, although it is close to altruism (helpful, true friendship, forgiving, and social justice) in the framework of the UHVs by Schwartz (1994) and Schwartz et al. (2012). Yitshaki and Kropp (2016) refer to a “vocation,” i.e., an inner direction of meaning. Weber (1958) wrote about a “divine inspiration” to do morally correct work. Even without divine inspiration vocation can be present, as an inner desire to serve others (Hall and Chandler, 2005). Divine inspiration did not emerge in the sample of this study, interviewees did however mention an inner desire to do something good for others. The fact that meaningfulness emerges in the results shows that this is an important driver for social entrepreneurs. This offers opportunities for better understanding of the social entrepreneur and his or her motivations by means of further research. It is possible that philosophical or ideological views play a role here, as various interviewees refer to this, e.g., religion and (political) worldviews.

For many entrepreneurs, a fundamental commitment to society plays a role in their motivation. For some, it is a remnant of education or early adulthood experiences, or later in their career path as a moment of insight under the influence of a life event. For others, being empathic and willing to serve the socially vulnerable seems to be an important character trait. This is also recognizable in the literature, for example, in Shumate et al. (2014) who mention “family legacy;” Saebi et al. (2018) who discuss a “prosocial personality” and Stephan and Drencheva (2017) who refer to higher scores on transcendental values. Social engagement appears to be a catch-all term, which invites for more detail in future research.

## Conclusion

Empathic involvement turns out to be an important driver for social entrepreneurs. About half of the 33 social entrepreneurs interviewed describe their motivations in a way that aligns with empathy. It can therefore be said that empathy is a driver of social entrepreneurship, but not for every social entrepreneur. For some of the interviewed entrepreneurs, there was a link between experiencing a transformative experience and the choice to dedicate their working life to a social enterprise. It is not always clear whether such a “life event” increases empathy, but it can be a reason to do things differently. A specific group of interviewees mentions a “life event” as a trigger for transformative change.

In addition to empathy and “life events,” other drivers have been found to be important for social entrepreneurs. For the majority of the interviewees, for example, their drive for self-realization plays a motivating role. In addition to self-realization, a sense of meaning and satisfaction appears to be an important motivator to become and remain a social entrepreneur. The assumption made in the literature, that empathy is the most important motivating factor of social entrepreneurs, does not hold. Other well-known motivators for doing business, such as self-realization, are found to play a motivating role as well. Nevertheless, empathy is an important driver for some of the interviewees. As a result, it is not easy to draw straight forward conclusions, but it is possible to sketch a nuanced picture of the various motives of social entrepreneurs. Empathy often plays a role, but not for every social entrepreneur. Sometimes empathy is awakened or reinforced by a life event, sometimes not. Even when empathy does not play a role, entrepreneurs can be motivated for social goals.

The limitations of this study provide possibilities for further research. First, in this study, social entrepreneurs were asked to talk freely about their experiences. Feelings of empathy were not explicitly mentioned in the interview questions, in order not to influence the participants by steering them into a certain direction. As a consequence, the conclusion that the role of empathy is less relevant for social entrepreneurs, because participants do not mention it explicitly, must be taken with caution. Furthermore, because of this procedure, statements of respondents cannot always uniquely be linked to (moral) empathy, and can sometimes also be associated with prosocial behavior which originates from sympathy or altruism. This issue should be kept in mind when interpreting the findings. Future studies about social entrepreneurs may focus on participants explicitly reflecting on their empathic feelings. Second, empathy is a driver for some social entrepreneurs, but it might be relevant to compare this with their “traditional” counterparts. There is a need to further investigate which personal motivations distinguish social entrepreneurs from traditional entrepreneurs (Lubberink, 2020). Several social entrepreneurs indicated that they made the switch to a social enterprise after disappointment or dissatisfaction with their job in the business world. A study of empathy in both groups can provide more insights into whether empathy is more important among social entrepreneurs.

## Data Availability Statement

The datasets presented in this article are not readily available because they are confidential. Requests to access the datasets should be directed to wim.lambrechts@ou.nl.

## Ethics Statement

Ethical review and approval was not required for the study on human participants in accordance with the local legislation and institutional requirements. Written informed consent for participation was not required for this study in accordance with the national legislation and the institutional requirements.

## Author Contributions

WL, MC, IM, and RO: conceptualization, formal analysis, and investigation. WL, IM, and RV: methodology. WL, MC, and IM: validation, data curation, writing – original draft preparation, writing – review and editing, and visualization. WL and MC: supervision. All authors contributed to the article and approved the submitted version.

### Conflict of Interest

The authors declare that the research was conducted in the absence of any commercial or financial relationships that could be construed as a potential conflict of interest.
